# Comparative efficacy of bariatric surgery for type 2 diabetes mellitus: A protocol for systematic review and network meta-analysis

**DOI:** 10.1097/MD.0000000000022755

**Published:** 2020-10-23

**Authors:** Xixiong Wang, Cunren Chen, Buping Zheng, Xiaolong Yang, Xiaoxin Zhang, Chenchen Yang

**Affiliations:** a Department of surgical oncology, Boao Evergrande International Hospital, Qionghai; b Department of Endocrinology, Hainan General Hospital, Hainan Affiliated Hospital of Hainan Medical University, Haikou, China.

**Keywords:** bariatric surgery, metabolic surgery, network meta-analysis, T2DM, type 2 diabetes mellitus

## Abstract

**Background:**

The comparative efficacy of bariatric surgical procedures for type 2 diabetes mellitus (T2DM) has not been completely elucidated. To investigate this question, we conduct a systematic review and network meta-analysis.

**Methods:**

The protocol followed preferred reporting items for systematic review and meta-analysis protocols (PRISMA-P) checklist. Two review authors will independently search the PubMed, Embase (Ovid), and the Cochrane Central Register of Controlled Trials databases. The primary outcome is T2DM remission. The secondary outcomes include BMI, HbA1c (%), and percentage excess weight loss (% EWL). Results from the network meta-analysis will be presented as summary relative effect sizes (WMD or RR) and relative 95% CIs for each possible pair of treatments. Outcomes will be combined based on different periods of follow-up (12 months, 36 months, and 60 months).

**Results:**

The results will provide useful information about the efficacy of bariatric surgical procedures in patients with T2DM.

**Conclusion:**

The findings of the study will be disseminated through peer-reviewed journal.

**INPLASY registration number:**

INPLASY202050053.

## 1 Introduction

Type 2 diabetes mellitus (T2DM) is recognized as one of the most challenging public health problems which greatly affects life expectancy.^[1,2]^ Medications for lowering glucose and lifestyle management typically result in a disease remission rate lower than 15%.^[3,4]^ Thus, bariatric surgery was investigated to improve glucose control and disease remission, and the efficacy was confirmed by several RCTs and meta-analyses.^[5–8]^ Therefore, bariatric surgery has been recommended as a treatment option for selected patients in several clinical practice guidelines and position statements.^[9]^

Surgeons continue to search for an ideal bariatric surgical procedure to help patients with T2DM control glucose levels and thus increase their life expectancy and improve their quality of life. Roux-en-Y gastric bypass (RYGB) and sleeve gastrectomy (SG) are 2 most common bariatric operational procedures used.^[10]^ Besides, adjustable gastric-banding (AGB), biliopancreatic diversion with duodenal switch (BPD-DS), greater curvature plication (GCP), and mini gastric bypass (MGB) have been introduced as alternative restrictive methods in metabolic surgery. However, most of those metabolic surgical procedures have never been compared with each other because of the lack of head-to-head trials and the limitation of traditional meta-analysis methods which could only conduct direct pairwise comparisons.^[11]^

Several previous network meta-analyses attempted to comparative efficacy of bariatric surgery for patients with T2DM.^[12–14]^ However, outcomes measured at different follow-up time points were combined which gives rise to heterogeneity.^[15]^ To provide concrete evidence for clinical practice, there is an urgent need for a thorough comparison of diabetes remission and cardiometabolic outcomes. Herein, we will conduct Bayesian network meta-analyses to investigate the questions based on different periods of follow-up and plan separate analyses.^[15]^ Besides, we will use risk ratio as measures of relative effect rather than odds ratio because misinterpretation of odds ratio usually overestimates the effect of the intervention.^[15]^

## 2 Method

Our study protocol has been registered in INPLASY.COM. The registration number is INPLASY202050053. The protocol followed preferred reporting items for systematic review and meta-analysis protocols (PRISMA-P) checklist.^[16]^ The systematic review and network meta-analysis will be planned and conducted adherence to Preferred Reporting Items for Systematic Review and Meta-Analysis Extension Vision statement (PRISMA-NMA).^[17]^ The study is a meta-analysis of aggregate data which do not involve human subjects and do not need ethical approval.

### 2.1 Eligibility criteria

#### 2.1.1 Types of study

Randomized controlled trails (RCTs) including T2DM patients receiving bariatric surgery in any type of procedures compared with each other or medical treatment will be eligible for inclusion. Only studies reported in English will be included.

#### 2.1.2 Patients and comparison of interventions

We will include studies that contain participants with a diagnosis of type 2 diabetes treated with at least 2 arms of following treatments: different bariatric procedures or medical therapy. There are no restrictions in age, ethnic distribution, and gender.

#### 2.1.3 Outcomes and measurements

The primary outcome is T2DM remission. The secondary outcomes include BMI, HbA1c (%), and percentage excess weight loss (% EWL). Studies reporting on at least 1 related outcome will be included. Continuous outcomes will be pooled with weighted mean difference (WMD). Dichotomous outcomes will be analyzed by calculating the relative risk (RR). Results from the network meta-analysis will be presented as summary relative effect sizes (WMD or RR) and relative 95% CIs for each possible pair of treatments. Outcomes will be combined based on different periods of follow-up (12 months, 36 months, and 60 months).

### 2.2 Data source

Two review authors will independently search the PubMed, Embase (Ovid), and the Cochrane Central Register of Controlled Trials databases using keywords and MeSH terms relating to bariatric surgery. We will scan the reference list of included studies or reviews identified through this search.^[18]^

### 2.3 Search strategy

Search strategy of PubMed was as follows:

#1 ((((((((((Bariatric Surgery) OR Gastric Bypass) OR Gastroplasty) OR Jejunoileal Bypass) OR Lipectomy)) OR “Roux-en-Y”)) OR “gastric banding”) OR “sleeve gastrectomy”) AND Diabetes
#2 ((((((((“Randomized Controlled Trial” [Publication Type]) OR “Controlled Clinical Trial” [Publication Type]) OR “randomized” [tiab]) OR “placebo” [tiab]) OR “Clinical Trials as Topic”[Mesh:NoExp]) OR “randomly” [tiab]) OR “trial” [ti])) NOT ((“Animals” [mh]) NOT “ humans” [mh])
#3 #1 AND #2

### 2.4 Study selection and data extraction

We will download and import the results of database search to EndNote Reference Manager Software (Clarivate Analytics, Philadelphia, Pennsylvania, USA) and remove duplicates. Then, citation titles and abstracts will be screened by 2 independent reviewers according to the eligibility criteria. Afterward, the full-text screen will be performed. We will describe the selection process using a PRISMA flow diagram.^[19]^

Two reviewers will independently use a priori designed data extraction form to collect data from the included studies.

The data variables to be extracted will include:

- Study characteristics including author name, journal, year of publication, country or region, and sample size.
- Eligibility criteria and baseline characteristics.
- Intervention characteristics including surgical procedures, medical treatment strategies, and duration and follow-up.
- Outcomes and measurements.

Any conflicts will be resolved by discussion with a third author.

## 3 Risk of bias assessment.

The risk of bias will be assessed using the Cochrane Collaboration's tool for randomized controlled trials by 2 reviewers independently.^[18]^ Disagreements will be resolved by discussion with a third author.

## 4 Data synthesis and statistical analysis

First, we will conduct traditional pairwise meta-analyses for all outcomes and comparisons at each time point by Stata (StataCorp, College Station, Texas). Q-test and *I*^2^ statistic will be used to estimate the heterogeneity among trials. When *I*^2^ index is less than 50% which indicates a non-statistical heterogeneity, a fixed-effects model will be used. On the contrary, if *I*^2^ ≥ 50%, we will investigate sources of heterogeneity. If no clinical heterogeneity exists, the random-effects model will be used. The publication bias will be assessed by funnel plot and Eggers test.

Then, we will perform Bayesian network meta-analyses to compare the efficacy of each surgical procedures using R x64 3.6.1 and Stata (StataCorp, College Station, Texas). We will assess inclusion and exclusion criteria as well as patient characteristics to transitivity assumption.^[20]^ Besides, we will use the node-splitting approach to evaluate inconsistency within each pair-wise comparison. We will present the Surface Under the Cumulative Ranking Area (SUCRA) values for each treatment to rank the different surgical procedures.^[11]^

## 5 Discussion

Metabolic surgery is an attractive option for patients with T2DM who have difficulty in making persistent efforts to make lifestyle changes for diabetes remission. However, which is the most efficacious surgical procedure for patients with T2DM is still uncertain regarding diabetes remission. Therefore, we conduct a network meta-analysis to investigate the question. To the best of our knowledge, this will be the first network meta-analysis conducted based on different periods of follow-up. We aim to summarize direct and indirect evidence and provide evidence-based suggestions for the clinical decision-making.

## Author contributions

**Conceptualization:** Xixiong Wang.

**Data curation:** Xixiong Wang.

**Formal analysis:** Buping Zheng, Xiaolong Yang.

**Funding acquisition:** Cunren Chen.

**Methodology:** Xixiong Wang.

**Software:** Xixiong Wang.

**Supervision:** Xixiong Wang, Cunren Chen.

**Writing – original draft:** Xixiong Wang, Xiaoxin Zhang.

**Writing – review & editing:** Xixiong Wang, Chenchen Yang.
